# Dermatology patient perspectives regarding consent, privacy, security, and identifiability of clinical photography at a tertiary cancer center: A qualitative study

**DOI:** 10.1016/j.jdin.2023.05.010

**Published:** 2023-05-27

**Authors:** Yuna Oh, Mary Sun, Lilly Gu, Trina Salvador, Liliane Sar-Graycar, Jennifer L. Hay, Elizabeth A. Quigley, Michael A. Marchetti, Allan Halpern, Veronica Rotemberg

**Affiliations:** a Dermatology Service, Memorial Sloan Kettering Cancer Center, New York, New York; b Behavioral Sciences Service, Memorial Sloan Kettering Cancer Center, New York, New York

**Keywords:** consent, perspectives, photographs, photography, qualitative

*To the Editor:* Despite widespread use of photography, data on patient attitudes towards photography is scarce^1^ and practices regarding use of photographs, consent guidelines, or deidentification are not standardized.^2^^,^^3^ We performed a qualitative study to elucidate patients’ experiences with photography and perspectives on photography consent, privacy, security, and identifiability.

After Institutional Review Board exemption from approval, English-speaking patients over the age of 18 undergoing in-room clinical photography (IRCP) or 3D-total body photography (3D-TBP) using Vectra WB360 system in the Memorial Sloan Kettering dermatology clinic from December 2019-September 2020 were eligible for enrollment. Participants were interviewed using a semi-structured guide, consisting of a loosely predetermined framework allowing for open-ended discussion, based on a framework by Kallio et al (Supplementary Fig 1, available via Mendeley at https://doi.org/10.1016/j.jdin.2023.05.010).^4^ Interviews were audio recorded and transcribed. Deidentified transcripts were coded, or segmented into themes or concepts, based on grounded theory (Fig 1).^5^ The codes were continuously refined until thematic saturation, or point at which no new themes or relationships between themes could be identified, and consensus were reached. Each interview transcript was analyzed for frequency of each code, for which inter-rater reliability using Cohen’s κ coefficient was 0.87.

**Fig 1:**
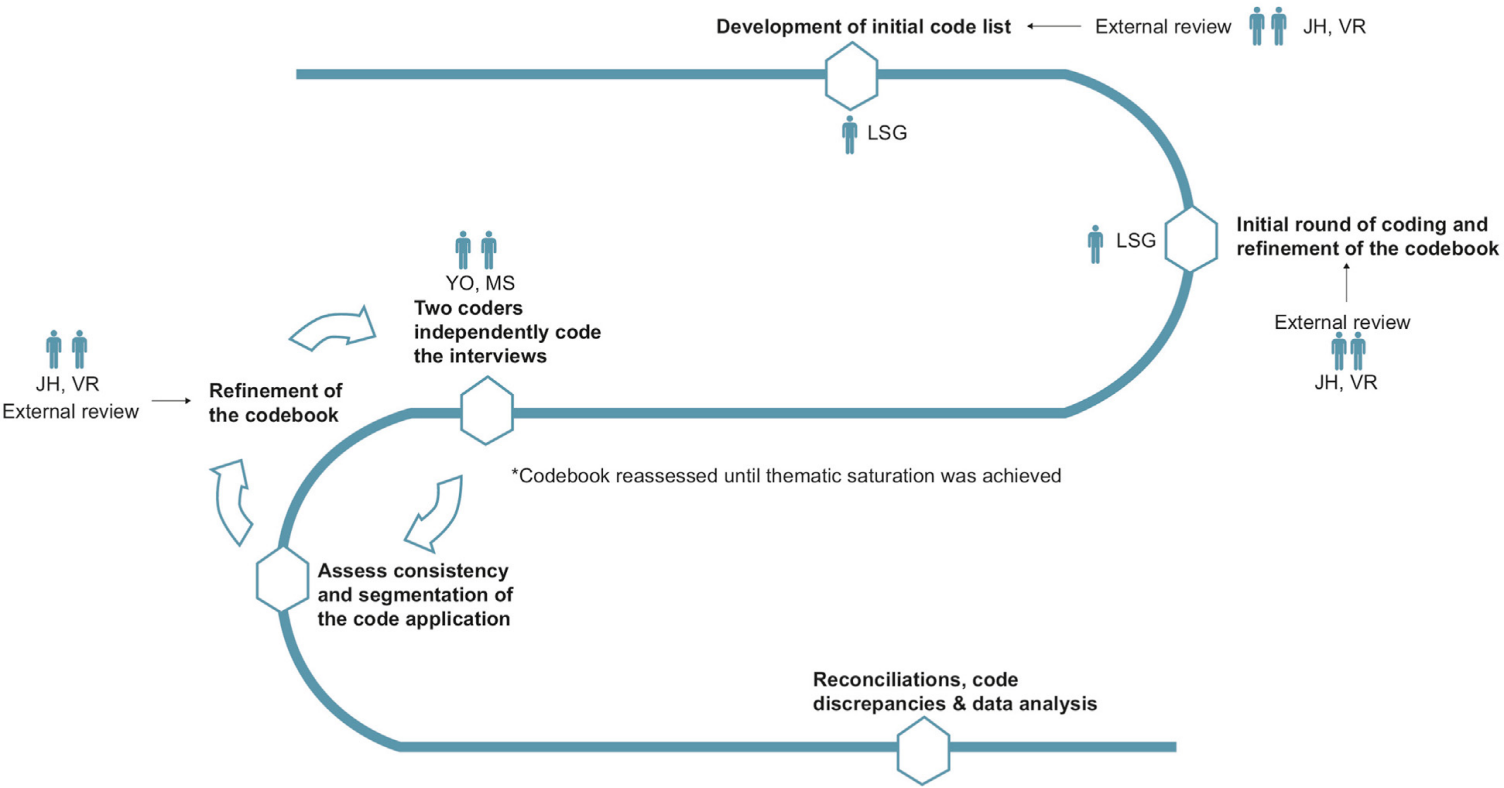
Development and implementation of the codebook. ∗JH: Jennifer Hay PhD; VR: Veronica Rotemberg MD, PhD; LSG: Liliane Say-Graycar MA; YO: Yuna Oh BS; Mary Sun BS.

Thirty-nine patients were approached, and 18 were recruited (49%) in 2 groups: 3D-TBP (*n* = 9) and IRCP (*n* = 9) (Table I). Median age was 59 (range 24-84). Five were male (28%) and 13 were female (72%). One participant self-reported as African American/Black, 1 Asian, and 16 White. Indications for photography included skin cancer history (*n* = 13, 72%), alopecia (*n* = 1, 6%), and rash (*n* = 4, 22%).

**Table I:** Patient characteristics

Characteristics	No. (%)		
	IRCP (*n* = 9)	3D-TBP (*n* = 9)	Overall (*n* = 18)
Age, mean (range), y	61 (29-84)	52 (24-74)	57 (24-84)
Sex			
Women	6 (67)	7 (78)	13 (72)
Men	3 (33)	2 (22)	5 (28)
Race			
African American or Black	1 (6)	0 (0)	1 (6)
Asian	1 (6)	0 (0)	1 (6)
White	7 (78)	9 (100)	16 (89)
Ethnicity			
Non-Hispanic	9 (100)	9 (100)	18 (100)
Hispanic	0 (0)	0 (0)	0 (0)
Clinical indications for photography			
Skin cancer	5 (56)	8 (89)	13 (72)
Melanoma	4 (44)	6 (67)	10
Non-melanoma skin cancers only	1 (11)	2 (22)	3
Rash (drug-related; cutaneous lymphoma)	3 (33)	1 (11)	4 (22)
Alopecia	1 (11)	0 (0)	1 (6)
Number of years since the most recent skin cancer			
Never	1 (11)	3 (33)	4 (22)
<1 y	1 (11)	4 (44)	5 (28)
1-5 y	3 (33)	1 (11)	4 (22)
>5 y	3 (33)	1 (11)	4 (22)
Unknown	1 (11)	0 (0)	1 (6)
# of 3D-TBP taken prior			
None	8 (89)	7 (78)	15 (83)
Once	1 (11)	2 (22)	3 (33)
Educational level			
High school diploma/GED	1 (11)	0 (0)	1 (6)
Some college, no degree	2 (22)	1 (11)	3 (17)
Completed college	3 (33)	3 (33)	6 (33)
Graduate or professional degree	3 (33)	5 (56)	8 (44)

*3-D*, 3-Dimensional; *GED*, general educational development; *IRCP*, in-room clinical photography; *TBP*, total body photography.

When asked about the reasons for photography, most participants (16, 89%) responded it was a routine part of care (Supplementary Table, available via Mendeley at https://doi.org/10.1016/j.jdin.2023.05.010). Other responses included physician recommendation (13, 72%) and diagnosis (11, 61%). No patients expressed concern about the consent process. Most participants were willing to allow nonclinical use of their photography: research/publication (15, 83%), teaching/education (14, 78%), and training artificial intelligence (14, 78%) but only if certain conditions were met: deidentification (11, 61%), removal of sensitive areas (7, 39%), or if separate consent was obtained (6, 33%). Almost all (16, 89%) considered face identifiable, but some also noted tattoos/piercings (14, 78%), scars (12, 67%), birthmarks (12, 67%), and jewelry (9, 50%) were identifiable.

Our study provides insight into patient perspectives on various aspects of photography. Although the experience was positive for most, some expressed concerns with sharing photographs with other providers and for nonclinical use of photographs. Patients preferred image access, particularly images containing identifiable or sensitive features, is limited to clinically necessary scenarios. For nonclinical use of photography, patients expressed need for separate consent. The wide variability in patient perspectives on “identifiability” highlights the need for consensus definition. Generalizability of the results is limited due to single-institution study design at a tertiary cancer center and low diversity in participants’ background. The findings of this exploratory study lay important groundwork for qualitative and quantitative studies, including ongoing quantitative studies evaluating patient and population-wide perspectives on clinical photography.

## Conflicts of interest

Yuna Oh: Dennis Gross Skincare Foundation Grant; Mary Sun: None; Lilly Gu: None; Trina Salvador: None; Liliane Sar-Graycar: None; Dr Hay: None; Dr Quigley: None; Dr Marchetti: None; Dr Halpern: Receiving personal fees from Canfield Scientific, SciBase, and Lloyd Charitable Trust and having an equity position with HCW Health and Skip Derm; Dr Rotemberg: Expert advising for Inhabit Brands, Inc.

## References

[1] Leger M.C., Wu T., Haimovic A. (2014). Patient perspectives on medical photography in dermatology. Dermatol Surg.

[2] Harting M.T., DeWees J.M., Vela K.M., Khirallah R.T. (2015). Medical photography: current technology, evolving issues and legal perspectives. Int J Clin Pract.

[3] Roberts E.A., Troiano C., Spiegel J.H. (2016). Standardization of guidelines for patient photograph deidentification. Ann Plast Surg.

[4] Kallio H., Pietilä A.-M., Johnson M., Kangasniemi M. (2016). Systematic methodological review: developing a framework for a qualitative semi-structured interview guide. J Adv Nurs.

[5] Foley G., Timonen V. (2015). Using grounded theory method to capture and analyze health care experiences. Health Serv Res.

